# Relationships between Food Insecurity, Self-Efficacy, and Changes in Body Mass Index among the Youth in Taiwan: Analysis from a Longitudinal Cohort Survey

**DOI:** 10.3390/children11060663

**Published:** 2024-05-29

**Authors:** Ya-Chi Huang, Chin Xuan Tan, Chih-Ting Lee, Meng-Che Tsai

**Affiliations:** 1 School of Medicine, National Cheng Kung University, Tainan 701, Taiwan; 2 Division of General Practice, Department of Medical Education, Changhua Christian Hospital, Changhua 500, Taiwan; 3 Department of Allied Health Sciences, Faculty of Science, Universiti Tunku Abdul Rahman, Kampar 31900, Malaysia; 4 Department of Family Medicine, National Cheng Kung University Hospital, College of Medicine, National Cheng Kung University, Tainan 701, Taiwan; 5 Department of Pediatrics, National Cheng Kung University Hospital, College of Medicine, National Cheng Kung University, Tainan 701, Taiwan; 6 Department of Medical Humanities and Social Medicine, College of Medication, National Cheng Kung University, Tainan 701, Taiwan

**Keywords:** food insecurity, weight status, self-efficacy, latent growth model

## Abstract

Background: Food insecurity is a heightened concern among economically disadvantaged youth, and it may contribute to the atypical body mass index (BMI) patterns frequently observed in this group. Self-efficacy seems to intervene in the negative impacts of contextual restraints. This study investigated the relationship between food insecurity, self-efficacy, and BMI trajectory among economically disadvantaged Taiwanese youth. Methods: We utilized three-wave longitudinal data from the Taiwan Database of Children and Youth in Poverty. The Food Insecurity Score (FIS) assessed food insecurity with a 4-item scale measuring reduced meal frequency, hunger, skipping meals, and economic constraints. Moreover, the General Self-Efficacy Scale (GSES) assessed self-efficacy, showcasing the ability to handle stress effectively and envision success scenarios, contributing to positive outcomes. By employing latent growth modeling, we were able to delineate the impacts of baseline food insecurity and self-efficacy on initial BMI and its subsequent growth trajectory. Results: Elevated baseline FIS significantly predicted higher initial BMI (coefficient = 0.420, *p* = 0.042). Baseline GSES was negatively associated with initial BMI (coefficient = −0.093, *p* < 0.001) but positively predicted the BMI growth rate (coefficient = 0.023, *p* = 0.011). Conclusion: Enhancing self-efficacy may be an effective multidisciplinary intervention to address psychosocial and socioeconomic factors when tackling weight problems in vulnerable youth groups.

## 1. Introduction

Food insecurity (FI) is usually defined as the uncertainty or inability to obtain sufficient food, and the affected individuals are unable to live a healthy life or feel unsatisfied. Despite the growth of Taiwan’s national wealth and food production, 7.8% of the population is reportedly low-fed or regularly hungry [1]. The intricate interplay between food insecurity, psychological factors such as self-efficacy, and obesity indices has attracted increasing attention, especially among economically disadvantaged youth [2]. The influence of household income on nutrition and weight status has been highlighted in the past research. Understanding this dynamic is crucial as it sheds light on how socioeconomic factors can shape diet patterns and ultimately contribute to the obesity epidemic among young people [3]. This highlights the complexity of the interaction between social determinants of health, such as food insecurity, and individual health outcomes, such as obesity. The increasing prevalence of overweight and obesity in this socioeconomically vulnerable population raises profound public health concerns [4,5]. 

The existing literature demonstrates a robust association between food insecurity and an elevated body mass index (BMI), shedding light on the malnutrition–obesity paradox [6]. The “malnutrition–obesity paradox” denotes the simultaneous occurrence of malnutrition and obesity within a population, household, or individual. It is prevalent in low-income, food-insecure households with limited access to nutrient-rich foods but abundant access to calorie-dense, processed foods. This situation leads to nutrient deficiencies and excess calorie intake, increasing the risk of undernutrition and obesity. Food insecurity is a known risk factor for obesity, particularly in socioeconomically disadvantaged populations. Understanding their relationships can inform the creation of multifaceted interventions targeting dietary behaviors and other health determinants. Longitudinal analyses can reveal temporal associations and potential causal pathways, crucial for effective preventive strategies. 

Moreover, the impact of food insecurity on weight gain trajectories may vary across different demographic groups. For example, a study found that the association between food insecurity and obesity was stronger among women compared to men [7]. This suggests that gender may moderate the relationship between food insecurity and BMI, highlighting the importance of incorporating intersectional perspectives in examining the link between food insecurity and weight outcomes. As cross-sectional evidence has laid the groundwork for understanding the link between food insecurity and BMI, longitudinal data offer a more nuanced understanding of the dynamic relationship between these factors. By examining how food insecurity influences weight gain trajectories over time and considering potential moderators such as gender, we can better delineate the causal pathways underlying the effect of food insecurity on weight gain trajectories. 

The role of self-efficacy, an individual’s belief in their ability to perform specific tasks or behaviors, has garnered significant attention in the context of weight management. Bandura’s Social Cognitive Theory postulates that self-efficacy serves as a pivotal determinant mediating health behaviors [8]. Numerous studies have demonstrated that higher eating self-efficacy, a domain-specific form of self-efficacy, is associated with successful weight loss, maintenance of a healthy weight, and the adoption of beneficial dietary and exercise practices among overweight/obese adults [9,10]. However, beyond domain-specific self-efficacy, general self-efficacy, which shapes the person’s agency and resilience, has not been thoroughly studied in relation to weight management. While existing research primarily focused on weight and obesity through the lens of task- or domain-specific self-efficacy, the role of general self-efficacy remains poorly examined. Some studies have suggested that general self-efficacy is related to characteristics associated with success in maintaining a healthy body weight [11,12,13,14], while others have found no significant relationship between general self-efficacy and body weight [15]. Elucidating the linkage between general self-efficacy and weight management could inform tailored interventions, particularly for disadvantaged youth facing psychosocial and socioeconomic adversities. Consequently, further investigation is warranted to elucidate the potential relationship between general self-efficacy and weight management. 

This study aimed to examine the predictive effects of food insecurity and self-efficacy on temporal changes in BMI measures among economically disadvantaged Taiwanese youth using three-wave longitudinal data. We further probed the interaction effect between food insecurity and self-efficacy on temporal changes in BMI to crystallize a more fine-grained understanding of this complex interplay. We formulated several hypotheses for this investigation. Firstly, we hypothesized that higher levels of baseline food insecurity will correlate with higher initial BMI levels and a more rapid increase in BMI over time. Secondly, we anticipated that higher levels of baseline general self-efficacy will be associated with initial BMI levels and a slower rate of BMI increase over time. Lastly, we proposed an interaction effect, suggesting that the relationship between food insecurity and BMI is moderated by levels of self-efficacy. These hypotheses will explore the complex dynamics of food insecurity, self-efficacy, and BMI. 

## 2. Methods

### Participants

Data were retrieved from the Taiwan Database of Children and Youth in Poverty (TDCYP), supported by the Taiwan Fund for Children and Families (TFCF). As described elsewhere, the TDCYP is a longitudinal study conducted since 2009 [1,5,16,17]. In brief, this survey was carried out on the subsidized families biennially until the subsidy receivers were disqualified from the allowance. The TDCYP used a multistage cluster sampling design to provide a nationwide representative cohort of economically disadvantaged families in Taiwan. Those who have dyslexia, cognitive impairment, or mental retardation diagnosed by medical professionals were excluded from the study. For this study, we used a subset of data on the 652 teenagers aged 12–18 years (in wave 3), completing all relevant items throughout the last three waves (wave 3 in 2013, wave 4 in 2015, and wave 5 in 2017) for this analysis. This study was approved by the Institutional Review Board of the National Cheng Kung University Hospital. 

## 3. Measures

### 3.1. Independent Variables

#### 3.1.1. Food Insecurity

In constructing the food insecurity scale (FIS), we adapted the Core Food Security Module devised by the United States Department of Agriculture, USDA (Nord and Hopwood, 2007 [18]), considering data availability in the TDCYP. Our 4-item scale encompasses multifaceted aspects of food deprivation over the past year: (1) Reduced meal frequency and snack intake; (2) Hunger due to lack of money; (3) Skipping breakfast or lunch to save money; and (4) Economic constraints in paying for lunch. Following precedented methods [17], we summed the dichotomized item scores to generate a composite FIS index ranging from 0 to 4, with higher scores denoting elevated severity of food insecurity.

#### 3.1.2. Self-Efficacy 

The General Self-Efficacy Scale (GSES) aims to provide a broad and stable assessment of an individual’s ability to deal effectively with various stressful situations. Students with a higher sense of self-efficacy are committed to challenging goals and often envision success scenarios [19] that contribute to positive behavior and outcomes [20]. Participants rated each item on a 4-point Likert scale ranging from 1 (not at all true) to 4 (exactly true). The items were: problem-solving ability, goal attainment, perseverance, confidence in unexpected situations, resourcefulness, investment of effort, calmness in difficulties, solution-finding, coping ability, and overall capability. The GSES score was computed by taking the mean of the 10 items, with higher scores indicating greater general self-efficacy. The reliability of this scale for wave 3 in our study showed Cronbach’s alpha = 0.91. 

### 3.2. Outcome Variables

#### Weight Status

Self-reported weight and height were applied to calculate the body mass index (BMI, kg/m^2^). BMI is commonly used to assess an individual’s weight status, used to decide whether it falls within a healthy range or whether they are overweight or obese [21]. The BMI assessment took place repeatedly in waves 3~5. Weight status was also considered in the subgroup analysis as a set of categorical outcome variables: underweight (<5th% of the same sex and age population), normal weight (between 5th% and 85th% of the same sex and age population), and overweight/obese (>85th% of the same sex and age population) [22]. 

### 3.3. Covariates

Gender (male vs. female), average household incomes (pretax earnings), and age at wave 3 (between 12 and 17 years) were treated as covariates. Growing up in low-income family units and communities poses numerous dangers and challenges to one’s well-being. Household production theory states that the assignment of parental assets shapes the well-being of children [23]. When family units encounter money-related strain and budgets are extended because of constrained income required for lodging, instruction, clothing, and well-being care, families are exceptionally likely to be prevented from devouring nutritiously satisfactory and secure nourishments or changing their dietary behaviors. All of these covariates were measured in wave 3. 

### 3.4. Statistical Analysis 

In the study, we used a structural equation modeling framework to analyze the longitudinal data. Initially, an unconditional latent growth model (LGM) was employed to estimate two latent factors, namely the intercept and the slope, using repeated measures of BMI values over three waves. Subsequently, a conditional LGM was constructed to examine the influence of self-efficacy and food insecurity on BMI, allowing us to simultaneously investigate the relationships between food insecurity, self-efficacy, and BMI changes. The final conditional model included an interaction term between food insecurity and self-efficacy as an independent variable. Given that individuals with different weight statuses may exhibit distinct growth trajectories, we stratified the analysis by weight status. Model fit was assessed using various indices. A good data-model fit was indicated by a root mean square error of approximation (RMSEA) < 0.05, Tucker–Lewis Index (TLI), and comparative fit index (CFI) > 0.99 [24,25,26,27,28]. LGM was conducted using AMOS 21 (SPSS Inc., Chicago, IL, USA) software, with additional analyses performed using SPSS 25.0 (SPSS Inc., Chicago, IL, USA).

## 4. Results

A total of 652 participants with a mean age of 14.89 (1.32) years (missing data of age = 0) and 342 (52.5%) girls (missing data of gender = 0) were enrolled in this study and their basic demographic data are described in Table 1. The average BMI increased with time, with an average of 20.85 (4.1) kg/m^2^ in wave 3, 21.54 (4.2) kg/m^2^ in wave 4, and 22.09 (4.4) kg/m^2^ in wave 5. Moreover, the average household income was NTD 3787.96/per person (missing data of gender = 0). 

When investigating individual effects of food insecurity and self-efficacy on the BMI trajectory (Table 2), we found that food insecurity was significantly associated with a higher initial level (coefficient = 0.420, *p* = 0.042) but not the growth of BMI. GSES was positively associated with the growth of BMI (coefficient = 0.023, *p* = 0.011) but negatively associated with the initial BMI level (coefficient = −0.093, *p* < 0.001). 

**Figure 1:**
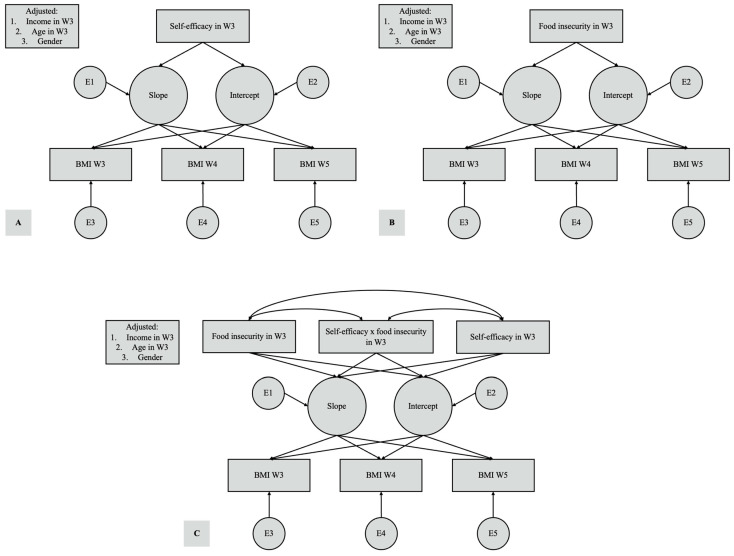
Interaction among adolescent BMI, General Self-Efficacy Score (**A**), food insecurity score (**B**), and the cross product of FIS and GSES (**C**) using latent growth modeling. E, measurement error term; W, wave.

To observe the effect of the interaction between FIS and GSES on BMI, we further included the cross-product of food insecurity and self-efficacy in the conditional LGM analysis (Figure 1C). The model showed a good fit, with a comparative fit index of 0.998, Tucker–Lewis index of 0.995, and RMSEA of 0.027 (90% confidence interval = 0.000–0.047) [29]. Table 3 showed Results of LGM on BMI and relationship with GSES, FIS, and the cross product of FIS and GSES. However, after adding the interaction product of food insecurity and self-efficacy, food insecurity did not affect the baseline or the changes in BMI values. On the other hand, self-efficacy was still associated with lower baseline BMI values in this model. The interaction term did not affect the BMI trajectory. 

Stratifying our conditional LCM analysis by weight status (Table 4), our findings indicated that only underweight individuals with higher levels of self-efficacy and food insecurity had lower initial BMI values. The interaction term was also significantly associated with the initial BMI values. These effects were not significant in the other two subgroups. 

## 5. Discussion

To our knowledge, our study was the first to describe the relationship between food insecurity, self-efficacy, and longitudinal BMI trajectory in a national sample of economically disadvantaged youth in Taiwan. First, we found that food insecurity was associated with higher BMI values at the beginning, but it could not explain the growth of BMI over time. Second, self-efficacy was negatively associated with baseline BMI value but positively associated with subsequent BMI growth. 

Collectively, these findings expanded on previous research on understanding the link between food insecurity and BMI over time. We found that food insecurity was associated with baseline BMI values. Like previous studies, our studies present a significant association between food insecurity and baseline BMI values [2,5,6]. In Wu’s study (2019), nearly three-quarters of the population surveyed reported experiencing at least one form of food insecurity, with each item of food insecurity being associated with a 45% higher probability of being obese. Our studies showed a trend between food insecurity and increased baseline BMI values, highlighting the magnitude of food insecurity in this part of the world that deserves public health attention. 

On the other hand, no significance was found in the association with the growth of BMI. This finding was contrary to the understanding that a higher food insecurity score was associated with a faster rate of increase in BMI over the subsequent years [4]. A previous study found that a higher food insecurity level in teenagers may lead to a faster growth in BMI, particularly in girls. However, in this study, food insecurity was not associated with the growth of BMI. We argue that food security may change over time due to a variety of external environments (economic conditions, government policies, interpersonal relationships, etc.) and personal factors (sports, self-perception, etc.) [30]. The population involved in our research is families that receive aid and financial support from our governmental agencies, so their food security status might have largely been ameliorated, so it has no impact on the growth of BMI during subsequent waves in our study. Therefore, it is imperative to consider strategies to decrease food insecurity during the critical developmental period of early adolescence, which may have positive long-term effects on subsequent weight control.

Our study found a negative correlation between the initial BMI and self-efficacy, inconsistent with previous results [15]. We have considered factors such as gender and income, previously thought to have an impact, as covariates for adjustment. Despite these adjustments, our study still found a significant relationship between BMI and self-efficacy. This finding indicated that BMI was significantly related to self-efficacy. Also, our study showed that the growth of BMI and self-efficacy were positively correlated in all subjects. 

Our findings have both positive and negative aspects. On the one hand, self-efficacy seems to affect body weight in this population negatively. Given that self-efficacy is associated with many important life outcomes, this result suggests that being overweight and obese may not be entirely independent of these life outcomes. In other words, self-efficacy is crucial for successfully performing various tasks and roles in life, and if we strive to promote self-efficacy, we can simultaneously change BMI and life outcomes. This is a good starting point for the government to invest more resources in improving self-efficacy in poverty-affected populations. 

On the other hand, people with lower self-efficacy may need to be educated to become more aware of this situation as it affects not only some important life outcomes but also their body weight status. If their problem of low general self-efficacy continues to worsen without intervention, they may develop long-term health problems and experience other harmful effects of low self-efficacy. 

While food insecurity significantly impacted baseline BMI, its effect was nullified after incorporating the interaction term between food insecurity and self-efficacy. On the contrary, self-efficacy maintained its significant predictive effect on baseline BMI despite including the interaction with food insecurity. Several reasons may account for the selective nullification of food insecurity. First, adding the interaction predictor may introduce multicollinearity that attenuates the sensitivity of the model in detecting the impact of food insecurity while sparing the effect of self-efficacy [31]. Second, variations in measurement and error distribution can also contribute to the discrepant degree of estimate alterations in the face of a complex modeling structure [32]. Compared to the subjective self-reported FIS, GSES as a psychometric instrument has undergone more stringent validity testing and demonstrated stability over time [33]. Hence, it may sustain significance even with extra adjustments. Taken together, statistical and methodological limitations provide plausible explanations for the unexpected finding that while the linkage between baseline FIS and BMI vanished, that of GSES persisted regardless of the interacting FIS. 

Further, we performed a subgroup analysis and divided the study subjects into three groups: normal weight, overweight/obesity, and underweight. Our study population had higher rates of overweight/obesity and underweight than the general population [22]. While FIS and GSES did not substantially affect baseline BMI or its growth trajectory in normal and overweight/obese groups, higher FIS or GSES corresponded to a lower BMI at baseline, specifically among underweight adolescents. In line with the food insecurity hypothesis [34], our results showed that higher levels of food insecurity, indicating higher instability in access to adequate food, led to lower baseline BMI among underweight disadvantaged adolescents. Facing threats of severe food shortage, those youths may restrict food intake or undertake other food conservation strategies as survival responses, resulting in undernutrition and weight loss. Also, this divergent phenomenon warrants deeper investigation into the psychological and motivational mechanisms underlying the perceptions of ideal weight and efficacy among underweight youth. 

One study proposed that underweight adolescents harbor a stronger motivation to obtain their personally defined “ideal weight” as they perceive more significant discrepancies from their goal states [35]. Therefore, those expressing higher confidence in their capabilities, as measured by the GSES, may more actively regulate their eating patterns in favored directions. Consequently, a higher GSES relates to a lower BMI, reflecting more progress toward self-determined ideal figures in this group [36]. Nonetheless, the conceptions of desired weight can differ by subgroups, as shaped by sociocultural influences. If underweight youths set lower target states through familial modeling or media exposure [35], higher perceived efficacy would be associated with lower matched goalposts. In other words, self-efficacy may partly capture subjective representations of ideal images alongside efficacy beliefs within each youth. Either way, elucidating group-specific psychological processes is imperative for accurately interpreting differential patterns and informing personalized interventions promoting healthy weight management across diverse adolescents. 

However, such adverse impact could be buffered when youths with high self-efficacy are exposed to food insecurity [37]. To actively understand and acquire food despite environmental constraints, food insecure youths high in self-efficacy are driven by growth needs to overeat and stockpile food when available. Such adaptive survival behavior consequently increases their baseline BMI. Our findings resonate with previous evidence that stress can prompt abnormal eating and weight fluctuations, especially among disadvantaged groups [38]. Believing in one’s competence to secure resources even in dire straits, the self-efficacious adolescents would go to great lengths to ensure a food supply. In light of the findings presented, future studies must continue to explore subgroup differences and investigate the potential mechanisms underlying the observed effects. This will not only increase our understanding of the factors that contribute to healthy weight management, but also provide crucial insights for designing effective interventions. Overall, the implications of this research are significant, as it offers insights that can contribute to the development of targeted interventions aimed at promoting healthy weight management in diverse populations.

These results must be interpreted cautiously, and several limitations should be considered. Three major limitations in this study could be addressed in future research. First, this study focused on gender, household income, and age, leaving other potential confounding factors uncontrolled. For instance, variables related to participants’ exercise habits are often lacking and, therefore, not controlled in the analysis (Petridou et al., 2019 [39]). To get a clearer view of BMI in socioeconomically disadvantaged populations, we may need more research focusing on these factors to tackle these issues adequately. 

Second, the Taiwan Fund for Children and Families provides subsidies based on household units. That is, adolescents who leave their original family may have been excluded from the sample. However, these underage populations who cannot receive financial assistance may represent a decent proportion of those who are potentially food-insecure after leaving home. Therefore, the results of our study may not apply to their situation. Due to the aforementioned factors and other data collection issues, a relatively small number of participants were assigned to the subgroups. Thus, the association analysis may not be robust enough to detect statistical significance. More research with a larger population is required to validate our results. 

Third, self-reported weights and heights may be biased, as lifestyles and eating behaviors usually change dramatically over puberty, within which this longitudinal study was conducted. Therefore, we should disseminate this study with more participants between the ages of 13 and 18 in more diverse groups to observe more of the impact on the general population. Despite this, the FIS we adapted still represents an overall severity of food insecurity [16], and its simplicity has merit, particularly in longitudinal epidemiological surveys where detailed information may not be available to build a more complex construct. 

Finally, little research focuses on self-efficacy in Taiwan, so most references are from abroad. However, cultural differences and other reasons influence self-efficacy, public perception of weight status, and their interaction [15]. We provide a first look at Taiwan in this issue; however, future research may be able to help determine how cultural factors influence the results found. Finally, the data collected are dated and may not reflect the present situation. Readers should be careful to take note of the time difference between the data collection and the proposed secondary data analysis. Despite this, the strength of our study is its longitudinal cohort study design, which collects prospective observation data throughout the entire adolescent period of the subject. Analyzing old archive data is probably an alternative approach to improving existing literature and inspiring new ideas [40].

## 6. Conclusions

Our study examined the relationship between food insecurity, self-efficacy, and weight status in economically disadvantaged youth in Taiwan. We found that food insecurity was associated with increased BMI values at the beginning but not the growth of BMI over time. Additionally, self-efficacy predicted a lower initial level but a faster growth of BMI over time. Our study provides insights that can contribute to developing targeted interventions to promote healthy weight management in diverse populations, especially among youth in poverty. We call for recognizing the interrelationship among food insecurity, self-efficacy, and their association with weight problems to diminish health inequalities in poverty when working with socioeconomically disadvantaged youth. A more comprehensive strategy, such as enhancing individuals’ food health concept and self-efficacy, should be exercised in more populations and may help reduce other mental or physical illnesses (ex., metabolic syndrome owing to obesity). 

## Figures and Tables

**Table 1:** Demographic information of participants.

	N (%) or Mean (SD ※)	Range
W3 age, years, mean (SD)	14.89 (1.315)	12 to17
Average household income (NTD/per person)	3787.96	0~17,900
Gender		
Female	342 (52.5%)	
Male	310 (47.5%)	
BMI		
Wave 3	20.85 (4.139)	11.42 to 45.71
Wave 4	21.54 (4.152)	13.70 to 40.90
Wave 5	22.09 (4.403)	10.28 to 42.09
Weight status in wave 3		
Overweight/obese	164 (25.1%)	
Underweight	65 (10%)	
Normal weight	423 (64.9%)	
Food insecurity scale		
0	241 (37.0%)	
1	291 (44.6%)	
2	111 (17.0%)	
3	7 (1.1%)	
4	2 (0.3%)	
General Self-Efficacy Scale	2.329 (0.559)	1.0 to 3.7

※ SD = Standard deviation.

**Table 2:** Results of LGM on BMI and its relationship with GSES and FIS, after adjustment for gender, age, and household income.

	Estimate	S.E. *	C.R. ^#^	*p*
Self-efficacy **→** Intercept	−0.093	0.028	−3.329	<0.001 *
Self-efficacy **→** Slope	0.023	0.009	2.53	0.011 *
Food insecurity **→** Intercept	0.420	0.207	2.032	0.042 *
Food insecurity **→** Slope	−0.036	0.067	−0.533	0.594

* S.E. = Standard error. ^#^ C.R. = Critical ratio.

**Table 3:** Results of LGM on BMI and relationship with GSES, FIS, and the cross product of FIS and GSES, with adjustment for gender, age, and household income.

	Estimate	S.E. *	C.R. ^#^	*p*
Self-efficacy **→** Intercept	−0.086	0.043	−2.022	0.043 *
Self-efficacy **→** Slope	0.026	0.014	1.832	0.067
Food insecurity **→** Intercept	0.488	0.875	0.558	0.577
Food insecurity **→** Slope	0.045	0.286	0.156	0.876
Self-efficacy × food insecurity **→** Intercept	−0.004	0.036	−0.122	0.903
Self-efficacy × food insecurity **→** Slope	−0.003	0.012	−0.257	0.797

* S.E. = Standard error. ^#^ C.R. = Critical ratio.

**Table 4:** Subgroup analyses based on varying BMI statuses.

	Estimate	S.E. *	C.R. ^#^	*p*
Normal group				
Self-efficacy **→** Intercept	−0.024	0.021	−1.139	0.255
Self-efficacy **→** Slope	0.019	0.013	1.429	0.153
Food insecurity **→** Intercept	−0.042	0.462	−0.091	0.928
Food insecurity **→** Slope	−0.188	0.294	−0.64	0.522
Self-efficacy × food insecurity **→** Intercept	0.007	0.019	0.376	0.707
Self-efficacy × food insecurity **→** Slope	0.007	0.012	0.545	0.586
Underweight group				
Self-efficacy **→** Intercept	−0.083	0.029	−2.896	0.004 *
Self-efficacy **→** Slope	0.007	0.029	0.256	0.798
Food insecurity **→** Intercept	−1.332	0.605	−2.202	0.028 *
Food insecurity **→** Slope	0.919	0.607	1.512	0.13
Self-efficacy × food insecurity **→** Intercept	0.05	0.024	2.086	0.037 *
Self-efficacy × food insecurity **→** Slope	−0.021	0.024	−0.889	0.374
Overweight/obese group				
Self-efficacy **→** Intercept	−0.149	0.077	−1.939	0.052
Self-efficacy **→** Slope	0.054	0.041	1.298	0.194
Food insecurity **→** Intercept	−1.268	1.338	−0.948	0.343
Food insecurity **→** Slope	0.322	0.723	0.445	0.656
Self-efficacy × food insecurity **→** Intercept	0.067	0.058	1.158	0.247
Self-efficacy × food insecurity **→** Slope	−0.02	0.031	−0.636	0.525

* S.E. = Standard error. ^#^ C.R. = Critical ratio.

## Data Availability

According to the ethical approval, the datasets analyzed during the current study are confidential and, therefore, not publicly available. The dataset can only be obtained from the corresponding author upon reasonable request.
